# Massachusetts Dental Society

**Published:** 1903-09

**Authors:** 


(To be continued.)

---

### MASSACHUSETTS DENTAL SOCIETY.

The thirty-ninth annual meeting of the Massachusetts Dental Society was held at the Massachusetts Charitable Mechanic Association Building, Boston, Mass., June 3 and 4, 1903, President Andrew J. Flanagan, of Springfield, Mass., in the chair.

*President Flanagan.*—I have great pleasure in introducing to you Dr. A. G. Minshall, of Northampton, who will read a paper entitled "The Influence of Diseases of the Air-Passages on the Teeth."

(For Dr. Minshall's paper, see page 670.)

### DISCUSSION.

*T. Morris Strong, M.D.,* Boston.—The concise paper which Dr. Minshall has just presented to the society leaves very little, if anything, to do except to commend it. I have often wished the positions were reversed and we had more dentists before our medical society instead of having physicians before the dental society, because, as a matter of fact, it is the family physician who is responsible for the continuation of a large part of these troubles. It is true there are men who will tell the parents, "Leave it alone, the child will grow out of it." Now, that is criminal advice, I do not care who gives it, and in this day, with the education that is going on in the two schools together, side by side, no man ought to give such advice as that. The parent will come in and say, "Well, doctor, what about the treatment in this case; will not medicine do something?" And my answer usually is that many of these conditions will disappear in time in certain cases. Whether they disappear on account of medicine or without any regard to the medicine, we do not know, but while they are disappearing they leave traces behind them which will never disappear, as in cases which have already been referred to by the essayist, in the alignment of the teeth, the general effect upon nutrition, the consequent decay of the constituents of the teeth, and the influence even on the respiratory tract, leaving the narrow jaw, the pigeon-breast, and the thousand and one ailments which follow obstructed nasal respiration. They do not, however, always disappear, for we will find the hypertrophied Luschka tonsil, for instance, in adults. I have removed them in persons between thirty-five and forty years of age where they were causing local trouble and interfering with respiration, so it does not necessarily follow that they will disappear.

Again we have two sources of trouble that we have to look out for. If the adenoids are present before the second dentition, and are removed, the trouble will be comparatively slight. A great many persons with the adenoids have no trouble with the shape of the jaw or the alignment of it, but a large proportion of the cases with adenoids that come to us in our clinics do have destructive conditions of the teeth themselves. How large the proportion is I am not prepared to say; I only know I have to do a great deal of talking to the patients to awaken them to the necessity of doing something for the teeth, and it rather surprised a great many of them even to-day to learn that anything can be done for the teeth so early; they think they should let them come out and others take their places. There again the family physician is at fault in not causing the patient to look out for the teeth. Of course, later, when they have perhaps escaped the attention of the family physician, they do come to you, and the application to make, from the information the essayist has given us, is that when these cases come to you you will be prepared to recognize the conditions and make the requisite examination. If they have been attended to you can go ahead with your direct special work, but first know whether they are there or not, and if they are still present remove them yourselves if you so desire. It is not a difficult operation. Of course, as you all know, it is a little bit bloody for a few minutes, but if you do not want to do it yourself, refer them back to the family physician, as a matter of courtesy perhaps, or to the specialist in this particular line of work. While they are there they do make trouble without any doubt. The adenoids, perhaps, are not the whole trouble, but they are the basis of it and they are there early in life; they are there at three months of age, and sometimes earlier. The young child comes along, has suffering respiration, cannot nurse, etc., and if you get your finger in the back of the throat you will find more or less of this tissue, and breaking it down gives relief to the nursing babe at once. But when these cases present, you should know whether they are there, and my point is that it is for you to consider and know this condition.

*George F. Eames, M.D., D.D.S.,* Boston.—I was very glad to hear reference made to the importance of the physician and the stomatologist coming together in meeting and discussing on common ground matters of medicine and dentistry. At the recent meeting of the American Medical Association, at New Orleans, action was taken which admits to full standing any graduate of a reputable dental college who is approved by the officers of the Section and receives a sufficient vote. As chairman of the Section on Stomatology, I most cordially invite you to attend upon the next meeting and become members of that Section. The meeting is at Atlantic City, easily reached and a delightful place in which to spend a few days.

The subject is a very important one, and has been very ably handled and discussed. I read a paper bearing on this subject in Baltimore in 1895, before the American Medical Association. I shall be glad to furnish any one with reprints of it. My views on this question are therein expressed in detail. The occasion of irregularities of the arch and teeth seem, from my point of view, to come not so much as a result of breathing through the mouth as the result of a general condition that coexists with mouth-breathing. I made some experiments at one time to show the pressure of the muscles of the mouth against the teeth when the mouth is open, as in mouth-breathing, and according to those tests there is practically no pressure made. This is in harmony with the anatomy of the parts. I do not believe that in mouth-breathing, in nine-tenths of the cases, the mouth is so widely open that the muscles involved are put upon the stretch so that pressure is brought to bear on the teeth. It is usually in sleep that the mouth is most widely open, and even then the relaxation of sleep allows the mouth to drop, and no force is exercised.

But I do believe, as the essayist has remarked, and which seems to be demonstrated, that obstruction coexists with the loss of development, for obstruction means a loss of function of any organ, and that tends to atrophy and waste of that organ.

It seems to me that the irregularities and other evils attendant upon adenoid growth are due in a considerable degree to a lack of nutrition, and that the high arch is not in reality a high arch, but apparently so; it is narrow rather than high, and, being narrow, appears to be high.

*Murdock C. Smith, D.D.S., M.D., D.M.D.,* Lynn.—Locality has a good deal to do with diseases of the air-passages. What would be considered good treatment in one locality would not do in another; patients living on the high dry hills of the western part of the State require different treatment to those living near the marshes along the sea-coast. A good deal of our trouble comes from patients who have consulted Boston practitioners, or physicians who were educated south of New York, who told them so and so. That may be all right for Boston or the South, but it will not do for Lynn.

In our work we get very little nasal obstruction in children; it is almost always adenoids; after puberty there may be a little deviation of the septum. If the adenoids are removed early in life it obviates the necessity of doing anything with the septum.

One of the best things a child can do who has difficulty in breathing is to push the little finger well up into the nose, thus enlarging the anterior cavity.

In my experience I have found that where there is deformity of the mouth due to trouble in the nose the mouth is of a different shape to where it is due to adenoids. Here are some casts of deformed mouths, and I will try to demonstrate the difference just referred to. Where it is due to adenoids there is seldom any lack of bony structure. Here (exhibiting cast) is a case where there is plenty of bone in the upper jaw, but it is in the wrong place. In young children with decayed temporary teeth always look for adenoids.

About puberty, when the arch is narrow, as shown by this cast (exhibiting cast), a good deal can be done, if there is not chronic hypertrophy of the turbinates, by widening the arch. It is no uncommon thing to widen the arch one-half inch, and most of the width comes in the median line, and it is not unreasonable to think that half of that distance is in the nasal fossa, making one-eighth inch on either side that would take the turbinates away from the septum enough to give room for good breathing.

Some of the Boston men teach that the antrum may be infected from the nasal cavity, or any of the neighboring cavities, and that in turn the antrum may infect the teeth so that diseases of the teeth may be secondary to diseases of the antrum. We can all agree that infection can spread from one sinus to another, but when it comes to infection from the antrum causing trouble with the teeth, it looks like an utter anatomical impossibility. Where the two diseases exist, if we look far enough back, we will find that in every case the disease started in the teeth, an alveolar abscess having started at the apex of the tooth and broken in the line of least resistance into the antrum.

Concerning the remarks of the gentleman who opened the discussion, that we should see that an operation had been or should be performed to remove the adenoids, it is not sufficient; make sure that they are removed. Unfortunately, they recur after some operators.

If the second gentleman that responded to the paper would take into consideration the action of the tongue, he would be better able to account for abnormal position of some of the teeth. The tongue has more to do with the alignment of the teeth than any other organ. When it is pushed well forward into the mouth, as in the act of running, to make room in the pharynx, it brings a tremendous pressure on the teeth; this is a point that is overlooked by both dentists and specialists.

(Exhibiting cast.) Here is a case of deformity due to adenoids that came on in the space of about one year in a boy of about fourteen years of age. The mouth was kept open all the time, and after the eruption of the second molars they kept on growing, and now there is no occlusion except on the molars.

(Exhibiting cast.) Here is a condition that it would be well to notice. Where you find the anterior teeth slightly separated and incrusted with tartar, and the only occlusion being on the first molars, you will find a child of rather low mental condition. In this case an operation for adenoids was recommended. Being the only grandson in the family, they would not consent. In a few years his mental condition became very poor, and he was taken to a specialist for feeble-minded children, who immediately recommended removal of the adenoids, and that was all the treatment he received. He went back to school and was able to keep up with the school-work for a year and then gradually failed. At one time he had tonsils which touched in the median line for fully three inches.

(Exhibiting cast.) Here is a case that is due to faulty breathing. In this case there is lack of bony growth, and it is a very rare case.

(Exhibiting cast.) Here is another case, a male, aged twenty-four; teeth worn away fully one-sixteenth of an inch; arch broad and well shaped; nasal obstruction. This abrasion is due largely to his efforts to keep the mouth closed to force air through the nose.

(Exhibiting cast.) A female, aged fourteen, with a deformity due to nasal obstruction, one side hypertrophied turbinate and the other a polypus; a good width to the arch.

In all these cases of narrow jaws you will find that adenoids, and adenoids only, are the cause of the trouble, and the sooner they are removed the better for your patient.

*Dr. Minshall* (closing).—I am sorry there was not more antagonistic criticism, so that one could have more to discuss, but there seemed to be a unanimity of opinion, at any rate, on the advisability of removing the adenoids and as to the effect they produce upon the teeth. The way they produce that effect is somewhat obscure.

Dr. Eames spoke of the deformity of the jaw, the narrowing of the high arch, etc., as having been due to the pressure of the muscles on the side. I did not put that as a factor myself, though I have heard it spoken of in that way, but you will also have to take into account the effect of atmospheric pressure; at first thought one would say that the pressure in the nose and mouth were equal, but that is not so, because the air in the nose when obstructed is more or less stagnant, and that in the mouth is not; in fact, there is a decided increase of pressure there from the strained inspirating efforts as producing a separate condition. And that is a very important fact.

Dr. Smith spoke of nasal obstruction from adenoids. I did not mean to distinguish those two at all. I think that nasal obstruction as caused by adenoids produces the same effect on the jaw as when due to other conditions. In many cases the passages have been entirely blocked by them. The effect of nasal obstruction, whether it is caused by polypus, hypertrophy, or by adenoids, is essentially the same.

The only possible additional factor which could arise in the case of adenoids is that sometimes the adenoids themselves, by their pressure on surrounding structures, diminish nutrition and the blood-supply of the bone while they are growing.

With regard to arching and narrowness, I cannot see that the breadth of the jaw is unaltered. I think in these high-arched palates you undoubtedly find a diminution of the distance there ought to be between the alveoli and that that has affected the palate, because in many of these cases you find the nasal septum bent over to one side, showing there is not sufficient room for proper growth owing to pressure from below; and the only reason that will give rise to that would be a higher elevation of the palate than normal.

We will probably all agree that the dentist and the physician should work together in these cases, and the result both to the physician and the dentist will be much more satisfactory than when these conditions are rectified before extensive changes in the bones have taken place.

